# Prolonged and Recurrent Intrahepatic Cholestasis of Pregnancy

**DOI:** 10.14309/crj.0000000000001182

**Published:** 2023-11-15

**Authors:** Rojin Kaviani, Daljeet Chahal, Michelle Ho Chung, Eric M. Yoshida

**Affiliations:** 1 Division of Gastroenterology, University of Alberta, Alberta, Canada; 2 Division of Gastroenterology, Division of Liver Transplant, Vancouver Coastal Health, Vancouver, British Columbia, Canada; 3 Department of Pharmacy, Vancouver Coastal Health, Vancouver, British Columbia, Canada

**Keywords:** intrahepatic cholestasis of pregnancy, case report, cholestasis, bile acid

## Abstract

Intrahepatic cholestasis of pregnancy is one of the most common disorders of pregnancy, which typically resolves in the postpartum period. Intrahepatic cholestasis is characterized by elevated bile acid levels that present as pruritus. The maternal clinical significance of recurrent and prolonged cholestasis is unknown. We discuss the longest reported case of postpartum cholestasis of 125 weeks.

## INTRODUCTION

Intrahepatic cholestasis of pregnancy (ICP) is a common disorder with adverse fetal outcomes affecting 0.3%–5.6% of pregnancies.^1^ It is characterized by pruritus on the palms and soles of feet with elevated serum bile acid concentration above 10–19 μmol/L.^1–3^ Symptoms can present up to 15 weeks before the development of ICP.^2^

ICP is a composite of genetic, hormonal, and environmental factors. The highest prevalence is in South American and Northern European populations.^1^ It presents in the third trimester because of rising levels of progesterone and estrogen metabolites that unmask ICP in genetically susceptible women and typically normalizes in 4 weeks postpartum.^4^ Given the observed seasonal variations and decline in prevalence over the past several decades, it is important to consider exogenous factors that also affect ICP development.^5^ Some studies attribute these findings to lower plasma selenium levels, reflecting lower soil concentrations throughout winter and improvements in diet.^6^

There is limited research on the outcomes of prolonged and recurrent cholestasis. Case reports of recurrent ICP are associated with primary biliary cholangitis and cirrhosis from unknown causes.^7–10^ We discuss the longest reported case of prolonged and recurrent ICP with postpartum bile acid elevation of 125 weeks.

## CASE REPORT

A healthy 40-year-old woman, Gravida 3 Para 3, was referred to our clinic for recurrent ICP. At age 35 years, she had her first pregnancy and developed bile acid elevation of 23.5 μmol/L (normal <10 μmol/L) at 38 weeks’ gestation. This prompted induction of labor, followed by an emergency cesarean section because of arrest at the second stage of labor without fetal complications. Bile acids were not measured postpartum.

**Figure 1.**
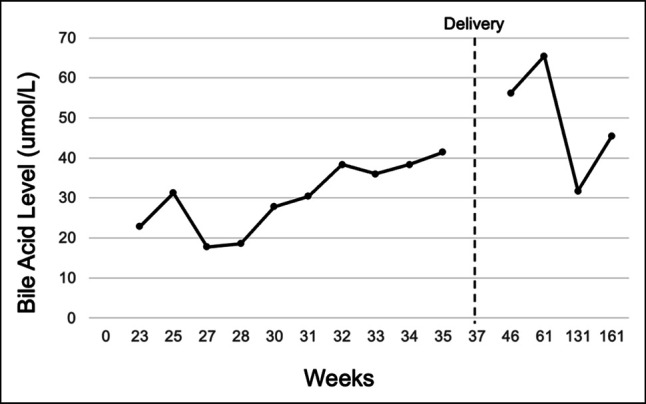
Cholestasis in the second pregnancy. Antepartum bile acid levels during the second pregnancy.

The patient had her second pregnancy 34 weeks later. Bile acid levels were 22.8 μmol/L when first measured at 28 weeks’ gestation (Figure 1). A planned C-section at 37 weeks resulted in a healthy infant. The bile acids continued to remain elevated for 125 weeks postpartum between 31.7 and 65.4 μmol/L, up until the third pregnancy. Bile acid levels were elevated during all trimesters of the third pregnancy, with a peak level of 41.8 μmol/L at 23 weeks’ gestation that decreased to 15.5 μmol/L at 34 weeks. An uncomplicated C-section was performed at 37 weeks. Presently at 12 days postpartum, bile acids have increased to 55 μmol/L.

The patient remained asymptomatic without features of decompensated liver disease throughout all pregnancies. The autoimmune review of systems was unremarkable. The patient does not consume alcohol or take prescribed medications. She is East Asian with no family history of liver disease or ICP. Physical examination was unremarkable. Complete blood count, electrolytes, renal function, liver enzymes, bilirubin, and international normalized ratio were normal throughout all pregnancies. Additional serology measured during the third pregnancy revealed a low-titer smooth muscle antibody at 1:20 (normal < 1:20) and positive Sjogren syndrome-A (SSA) antibody at > 8 U (normal < 1 U). The anti-mitochondrial antibody, nuclear antibody, deoxyribonucleic acid double-strand antibody, Sjogren syndrome-B antibody, scleroderma-70 antibody, Smith antibody, and immunoglobulin levels were negative. Ferritin, thyroid-stimulating hormone, hemoglobin A1c, ceruloplasmin, α-1 antitrypsin, and lipid levels were normal. She was immune to hepatitis A and B with no hepatitis C or human immunodeficiency virus infection. A fasting transient elastography (TE) at 6 weeks’ gestation of the third pregnancy revealed a liver stiffness level of 9.4 kPa (normal < 7.3 kPa) and Controlled Attenuation Parameter Score of 228 dB/m. Given her asymptomatic presentation, liver biopsy and ultrasound were not performed. The patient was diagnosed with prolonged ICP. She was followed clinically to monitor for the development of systemic disease without treatment. Owing to the positive SSA antibody, there was enhanced fetal monitoring throughout the third pregnancy.

## DISCUSSION

We present the longest case of postpartum elevated bile acid levels of 125 weeks reported globally to date. Maternal and fetal outcomes were unremarkable in our case, signifying that there are asymptomatic presentations of prolonged bile acid elevation that do not require invasive investigations. It is prudent to identify patients who require additional tests, such as liver ultrasonography and viral hepatitis serology, by recognizing atypical features of ICP.^2,3^ Atypical features are early onset, severe symptoms, markedly abnormal transaminases, and features of acute liver failure.^2,3^ Risk factors of ICP include a history of contraceptive-induced cholestasis, advanced maternal age, and personal and family history of ICP.^2,3^ Bile acid levels > 40 μmol/L identify patients at higher risk of fetal complications.^1,2^ Levels > 100 μmol/L are associated with a higher risk of stillbirth.^1,2^

When evaluating ICP, additional investigations with routine imaging or serology are not recommended because it typically resolves without complications or discovery of underlying liver disease.^2,3^ However, large population-based studies in Northern Europe have found associations between ICP and the development of cardiovascular disease, hepatitis C, autoimmune hepatitis, hepatobiliary disease, non-alcoholic pancreatitis, and malignancy.^11–13^ There was also a higher incidence of preeclampsia and gestational diabetes in women with ICP.^2^ In our case, the increased liver stiffness on TE is challenging to interpret because values can increase with pregnancy progression.^14^ TE values are dependent on the operator and whether it was performed in a fasting state. Without features of autoimmune hepatitis and liver disease, the significance of a smooth muscle antibody at the upper limit of normal and SSA antibody positivity is uncertain. Although a positive SSA antibody has a prognostic value for the development of Sjogren syndrome, its significance lies in its association with the development of neonatal lupus and congenital heart block.^15^

Management is expectant because it remains controversial whether treatment alters maternal or fetal outcomes.^5^ For severe pruritus, ursodeoxycholic acid at 10–15 mg/kg provides symptomatic benefit more effectively than steroids or cholestyramine.^2,3,5^ Definite management includes delivery at 37 weeks’ gestation to prevent fetal complications.^2,3^ Notably, ICP is not a contraindication to future pregnancies.^2^

Our case has multiple abnormal features; however, there is no consensus among guidelines on the frequency of investigations and duration of follow-up in ICP because of the lack of evidence in its interpretation.^5^ The clinical significance of prolonged and recurrent ICP is unknown, and further long-term research is required to evaluate maternal outcomes and the development of liver disease.

## DISCLOSURES

Author contributions: R. Kaviani and EM Yoshida made a substantial contribution to the concept or design of the article or acquisition, analysis, or interpretation of data for the article and drafted the article or revised it critically for important intellectual content. D. Chahal drafted the article or revised it critically for important intellectual content. All authors approved the final version to be published. EM Yoshida is the article guarantor.

Financial disclosure: There are no direct conflicts of interest. Possible conflicts outside of this case report include an author of this paper is an investigator of clinical trials sponsored by Intercept, Madrigal, Genfit, Novodisk, Pfizer, Gilead Sciences, Allergan, Celgene, Sonic Incites. An author has received honoraria for CME lectures sponsored by Intercept Canada and received unrestricted research grant from Paladin Laboratories.

Informed consent was obtained for this case report.
